# Glycosylphosphatidylinositol Anchors from Galactomannan and GPI-Anchored Protein Are Synthesized by Distinct Pathways in *Aspergillus fumigatus*

**DOI:** 10.3390/jof4010019

**Published:** 2018-01-23

**Authors:** Jizhou Li, Isabelle Mouyna, Christine Henry, Frédérique Moyrand, Christian Malosse, Julia Chamot-Rooke, Guilhem Janbon, Jean-Paul Latgé, Thierry Fontaine

**Affiliations:** 1 Unité des Aspergillus, 25 rue du Docteur Roux, Institut Pasteur, 25 rue du Docteur Roux, 75015 Paris, France; lee19910503@gmail.com (J.L.); imouyna@pasteur.fr (I.M.); chenry@pasteur.fr (C.H.); jplatge@pasteur.fr (J.-P.L.); 2 Unité de Biologie des ARN des Pathogènes Fongiques, Institut Pasteur, 25 rue du Docteur Roux, 75015 Paris, France; frederique.moyrand@pasteur.fr (F.M.); guilhem.janbon@pasteur.fr (G.J.); 3 Unité de Spectrométrie de Masse pour la Biologie, Institut Pasteur, CNRS USR 2000, 28 rue du Docteur Roux, 75015 Paris, France; cmalosse@pasteur.fr (C.M.); julia.chamot-rooke@pasteur.fr (J.C.-R.)

**Keywords:** glycosylphosphatidylinositol, *Aspergillus fumigatus*, *PER1*, cell wall, galactomannan

## Abstract

Glycosylphosphatidylinositols (GPIs) are lipid anchors allowing the exposure of proteins at the outer layer of the plasma membrane. In fungi, a number of GPI-anchored proteins (GPI-APs) are involved in the remodeling of the cell wall polymers. GPIs follow a specific biosynthetic pathway in the endoplasmic reticulum. After the transfer of the protein onto the GPI-anchor, a lipid remodeling occurs to substitute the diacylglycerol moiety by a ceramide. In addition to GPI-APs, *A. fumigatus* produces a GPI-anchored polysaccharide, the galactomannan (GM), that remains unique in the fungal kingdom. To investigate the role of the GPI pathway in the biosynthesis of the GM and cell wall organization, the deletion of *PER1*—coding for a phospholipase required for the first step of the GPI lipid remodeling—was undertaken. Biochemical characterization of the GPI-anchor isolated from GPI-APs showed that the *PER1* deficient mutant produced a lipid anchor with a diacylglycerol. The absence of a ceramide on GPI-anchors in the *Δper1* mutant led to a mislocation of GPI-APs and to an alteration of the composition of the cell wall alkali-insoluble fraction. On the other hand, the GM isolated from the *Δper1* mutant membranes possesses a ceramide moiety as the parental strain, showing that GPI anchor of the GM follow a distinct unknown biosynthetic pathway.

## 1. Introduction

Glycosylphosphatidylinositols (GPI) are specific glycolipids that allow attachment of soluble proteins at the outlet layer of the plasma membrane. GPI-anchored proteins (GPI-AP) are ubiquitously found in eukaryotic organisms and are involved in several biological functions such as ligand recognition, enzymatic activities, cell–cell interaction, and host defense [1]. The sequence of GPI-APs is characterized by the presence of a hydrophobic peptide signal for GPI anchoring at the C-terminal. Using an in silico analysis, 115 GPI-APs have been predicted in *Aspergillus fumigatus* [2]. In the yeast model *Saccharomyces cerevisiae*, a number of GPI-APs are required for organization and remodeling of the cell wall, making the GPI biosynthesis an essential pathway to fungal life and morphogenesis. The fungal cell wall is mainly composed of polysaccharides, chitin, α and β-glucans, galactomannan, and galactosaminogalactan, organized in a three-dimensional network [3]. In addition to GPI-anchored polysaccharide hydrolases such as chitinases and glucanases, three specific GPI-anchored transglycosidase activities are required for the building of the cell wall skeleton: (i) Two types of β-1,3-glucan remodelase have been characterized first in *A. fumigatus* [4,5]: the β-1,3-glucan branching enzyme and the GAS/GEL family members responsible for β-1,3-glucan chain elongation. These β-1,3-glucanosyltransferase activities are essential for the biosynthesis of the fungal cell wall [6,7,8]; (ii) The Crh family (for congo red hypersensitive) contains putative transglucosidase activity involved in the β-glucan-chitin reticulation [9]. Five orthologs have been identified in *A. fumigatus*, but their function remains unknown; (iii) The GPI-anchored Dfg (Defect in filamentous growth) family is composed of two proteins in yeast with redundant activities. Double knockout was synthetically lethal [10]. In *A. fumigatus*, seven orthologs have been identified and are required for the galactomannan cross-linking onto the cell wall β-1,3-glucan [11].

**Figure 1:**
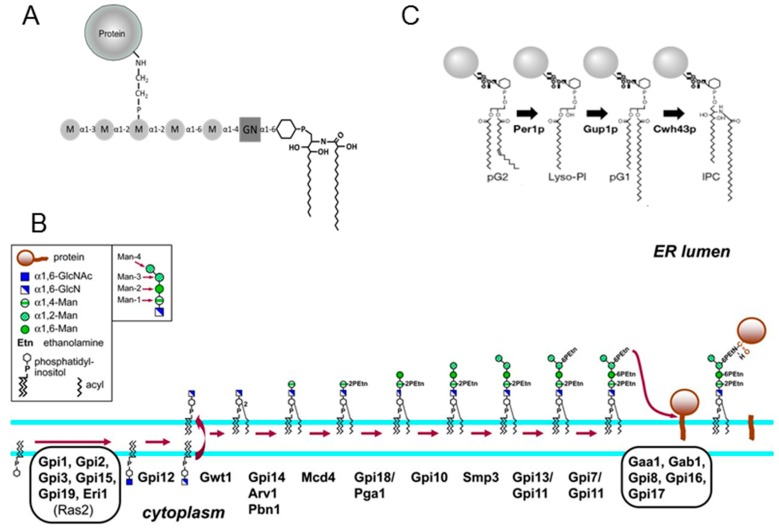
(**A**) Scheme of Glycosylphosphatidylinositol (GPI) structure from GPI-anchored proteins (GPI-Aps) in *A. fumigatus*; (**B**) Scheme of the biosynthesis of the GPI precursor and its transfer to protein in the ER membrane in fungi. GlcNAc addition to PI and de-N-acetylation of GlcNAc-PI to GlcN-PI occur at the cytoplasmic face of the ER membrane, and further additions to the GPI occur on the lumenal side of the ER membrane [16] (reprinted with the permission from Genetics Society of America). (**C**) Scheme of lipid remodeling of the GPI pathway in fungi. pG1 and pG2: phosphodiacylglycerol, IPC: inositolphosphoceramide, M: mannose, GN: glucosamine.

Fungal GPI-anchors are composed of a common glycan structure containing 4 mannose residues linked to a glucosamine. The anchored protein is linked to the third mannose residue of GPI through a phosphoethanolamine bridge and one more mannose residue may be added to the common core [12,13] (Figure 1). The second particularity of fungal GPI is based on the lipid moiety that is an inositol-phosphoceramide [13,14]. In addition to GPI-APs, *A. fumigatus* produces a lipogalactomannan (LGM) which is, to date, the sole fungal GPI-anchored polysaccharide [15]. The LGM is constituted by the elongation of the mannan moiety of GPI structure. Interestingly, the mannan chain of the GM is composed of repeat units of 4 mannose residues with 3 α-1,2 and 1 α-1,6 linkage similar to the common structure of fungal GPI. However, one question remains open: does the LGM follow the GPI-pathway of GPI-APs?

The GPI biosynthesis is a specific pathway that occurs at the endoplasmic reticulum (ER) membrane. Nine biochemical steps are required for the complete synthesis of the intermediate prior to being transferred to the target protein: transfer of *N*-acetylglucosamine (GlcNAc) onto a phosphatidylinositol (PI), GlcNAc deacetylation, inositol acylation, and the addition of 4 mannose and 3 phosphoethanolamine residues (Figure 1) [17]. Apart from the phosphoethanolamine transfer onto the second mannose, all steps are essential to fungal growth [17]. This pathway is conserved in filamentous fungi, including in *A. fumigatus* [18]. After the transfer to the protein bearing the C-terminal signal sequence for GPI attachment and before the exit of ER, a GPI remodeling occurs to modify the lipid moiety and to remove the first two phosphoethanolamine groups [19,20]. Finally, the last modification of the GPI-anchor in *A. fumigatus* is the addition of the fifth mannose by a Golgi α-1,3-mannosyltransferase [21].

Fungal lipid remodeling is specific and has been well studied in *S. cerevicsiae*. After the inositol deacylation by Bst1p that occurs in all eukaryotic cells, the GPI fungal lipid remodeling leads to the substitution of the diacylglycerol by a ceramide [22,23] and occurs in three steps: de-*O*-acylation in the sn-2 position of the glycerol by the phospholipase A2 activity, Per1p [24]; then re-*O*-acylation by Gup1p that transfers a long chain fatty acid [25]; and the substitution by a ceramide by Chw43p [26] (Figure 1). These lipid remodeling events are not essential for fungal life, but essential for the cellular trafficking of GPI-APs, their association to lipid microdomains, and their localisation [14,25,27]. The importance of this lipid remodeling is poorly investigated in filamentous fungi. Only *PER1* has been deleted in *A. fumigatus* [28]. The deletion of *PER1* has shown that the lipid remodeling of GPI is required for normal growth, conidiation, and full virulence. In this study, we took advantage of the non-essentiality of the GPI-lipid remodeling to investigate the role of the GPI pathway in LGM biosynthesis and its incorporation into the cell wall. Our data demonstrate that GPI anchors from GPI-APs and LGM follow two different biosynthetic pathways.

## 2. Materials and Methods

### 2.1. Growth Conditions

Parental (*∆ku80*) and mutant (*∆per1*) strains of *A. fumigatus* were grown at 37 °C in either *Aspergillus* minimal medium (AMM) containing 1% glucose and 5 mM ammonium tartrate, or Sabouraud (2% glucose, 1% Mycopeptone, Difco BD, Le Pont de Claix, France), or 2% Malt (Cristomalt). Media were either liquid or supplemented with 2% agar. When necessary, 6% KCl was added to solid media to enhance conidiation. Conidia were collected from agar media plates after 10 days of growth at 37 °C, using water solution containing 0.05% Tween 20.

### 2.2. Construction of the Δper1 Mutant

First, the plasmid pNE476 was constructed by cloning the hygromycin marker amplified from the plasmid pAN7-1 in the pGEMT easy vector [29]. The pNE478 plasmid was then constructed by cloning the PCR amplified fragment containing the GFP gene at the pnE476 BglII/BamHI sites. The disruption cassette was then constructed using PCR fragments amplified from either the plasmid pNE478 or the genomic DNA extracted from the strain *∆ku80* [30], following a strategy previously applied to *Neurospora crassa* deletion cassettes [31]. The *A. fumigatus* strain *∆ku80* was then transformed by electroporation and transformants were screened on complete medium supplemented with 100 μg/mL hygromycin (Sigma, Saint Louis, MO, USA). The correct deletion of the gene was tested using primers within and outside the cassette. The absence of additional ectopic integration of the cassette was checked by Southern blot experiment (Table S1). Transformants obtained were analyzed by PCR and Southern blot analysis using the DIG probe protocol (Roche Diagnostics, Mannheim, Germany).

### 2.3. Fungal Morphotype of the Δper1 Mutant Strain

The fungal growth of the different strains was measured on solid medium after 48 h of incubation at 37 °C or 50 °C. Growth in Sabouraud liquid culture was investigated using flasks shaken at 150 rpm at 37 °C. Mycelium morphology was observed by optic and fluorescence microscopy in the presence of 1 µg/mL calcofluor white. Dry weights were taken after 24 h of growth. The conidiation rates were estimated by inoculation of conidial suspensions (150 µL, 10^5^/mL) into three tubes of Malt agar. After 1 day at 37 °C and 6 days at 25 °C, conidia were recovered with 4 mL water containing 0.05% Tween 20, filtered on membrane (cell strainer, 40 µm) and counted using haemocytometer. Survival of 40-day-old conidia on Malt-agar slant at room temperature was estimated by the quantification of conidia germination on Sabouraud agar medium for up to 12 h at 37 °C.

The susceptibility of the mutant strains with regards to cell wall disturbing compounds or antifungal drugs was estimated by using ten-fold dilutions of conidia, starting at 10^6^ spores as the highest concentration, spotted (5 µL) onto plates containing calcofluor white (40 µg/mL), Congo red (50 µg/mL), or SDS (0.01%). Plates were incubated for 72 h at 37 °C in a humid atmosphere.

Permeability of the conidia to FITC was investigated by incubating 200 µL of an aqueous suspension of 2-week-old conidia (2 × 10^7^ conidia/mL) with 30 µL of FITC solution (0.2 mg/mL in Na_2_CO_3_ 0.1 M pH 9) during 3 h at room temperature in darkness. The conidia were washed three times with 0.05% Tween 20 water before observation under fluorescent light at 518 nm.

### 2.4. Microscopy

Mycelium and conidia were observed by optic and fluorescence microscopy. Images were recorded with an Evos FL apparatus (Life Technologies, Marly le Roi, France). Wavelengths used for fluorescence were λex 470/22 nm and λem 510/42 nm for FITC labelling and λex 357/44 nm and λem 447/60 nm for calcofluor white labelling.

### 2.5. Carbohydrate Analysis of the Cell Wall and Culture Supernatant Fractions

After 24 h of growth in shaken Sabouraud liquid medium at 37 °C, 150 rpm, mycelia and culture supernatant were separated by filtration. Macromolecules from the supernatant were precipitated by 3 volumes of ethanol at 4 °C overnight and collected by centrifugation (5 min, 4000× *g*). Cell wall fractions (alkali-soluble and alkali-insoluble fractions) were obtained after mycelium disruption and centrifugation as previously described [32]. Polysaccharides from the cell wall were separated by function of their alkali-solubility [32]. Neutral hexoses were estimated by the phenolsulfuric method using glucose as standard [33]. Osamines were quantified by HPLC after acid hydrolysis by 6 N HCl at 100 °C for 6 h [34]. Proteins were quantified by the BCA assay (ThermoScientific, Rockford, IL, USA) using BSA as standard. Monosaccharides were identified and quantified by gas-liquid chromatography (GLC) after acid hydrolysis with 4 N trifluoroacetic acid at 100 °C for 4 h [32]. Branching level of β-1,3-glucan from the cell wall alkali-insoluble fraction was estimated by HPLC after laminarinase-A digestion. Prior to laminarinase digestion, 5 mg of the AI fraction was treated by 100 mM *m*-IO_4_Na (1 mL) at room temperature in the dark for 3 days. After the addition of 20 µL, the AI fraction was sequentially washed with water, reduced in the presence of 10 mg/mL BH_4_Na overnight, neutralized by addition of acetic acid, washed with water, treated by 10% acetic acid at 100 °C for 1 h, and washed with water. Enzymatic digestion and the HPLC procedure have been previously described [4].

### 2.6. GPI-AP Purification and Detection

In order to increase the biomass production, mycelium was grown in Sabouraud medium using a 1.5 L fermentor for 24 h as previously described [15]. After mycelium disruption, the whole cellular membrane was isolated by ultracentrifugation [13]. Total membrane proteins were solubilized with 2% Triton X100, 5 mM HgCl_2_ as PI-PLC inhibitor and anti-protease cocktail (Complete mini EDTA-free, Roche Diagnostics, Mannheim, Germany) in TrisHCl 50 mM, EDTA 2 mM pH 8.8 buffer using a Dounce homogeinezer (Wheaton, Millville, NJ, USA) for 15 min in ice [13]. After ultracentrifugation (89,400× *g*, 30 min), the supernatant was diluted 4 times with TrisHCl/EDTA pH 8.8 buffer and applied onto a Q-Sepharose column (2.2 × 7 cm, GE Healthcare, Uppsala, Sweden) equilibrated in 0.05% Triton X100, 0.5 mM HgCl_2_ in TrisHCl/EDTA pH 8.8 buffer at the flow rate of 15 mL/h. After elution of the unbound fraction, proteins were eluted by a NaCl gradient (0 to 500 mM, 200 mL) in the same buffer. A second anion exchange chromatography step was used to purify GPI-APs on a DEAE-FastFlow column (5 mL, GE healthcare, Uppsala, Sweden) equilibrated in 20 mM TrisHCl pH 7.5 with 0.05% Triton X100 and 0.5 mM HgCl_2_ at 1 mL/min. Proteins were eluted with a linear gradient of NaCl (0 to 400 mM, 60 min). Prior to analysis of the GPI anchor, fractions containing GPI-APs were boiled for 10 min to inactivate endogenous protease and PI-PLC activities. Proteins were detected and quantified by the bicinchoninic acid method (BCA protein assay kit, Pierce, ThermoScientific, Rockford, IL, USA). Phosphatase activity was detected using the *p*-nitrolphenyl-phosphate substrate. GPI-APs were visualized by Western blot after exogenous PI-PLC digestion and detection with an anti-CRD mouse antibody as previously described [13]. GPI-APs from *A. fumigatus*, i.e., Gel4p, Ecm33p and PhoAp, were also detected by Western blot using mouse antibodies [6,7,35,36].

### 2.7. Lipogalactomannan (LGM) and Glycosylinositolphosphoceramide (GIPC) Purification

The LGM and GIPC were purified from preparation of the whole membrane as previously described [15,37]. Briefly, total lipids including GIPC were extracted by chloroform/methanol/water (10/10/3) extraction. After evaporation of organic solvents, GIPCs were purified by a butanol/water partition. Insoluble fraction containing LGM after chloroform/methanol extraction was digested by protease from *Streptomyces griseus* (Sigma, Saint-Louis, MO, USA) at 37 °C in 50 mM TrisHCl pH 7.5 buffer with 5 mM sodium azide for 2 days. Peptides were removed by 30% propanol-1 extraction and dialysis. Then, LGM fraction was purified on an Octyl-Sepharose column (1.6 × 20 cm, Sigma, Saint Louis, MO, USA) equilibrated with 5% propanol-1, 100 mM NH_4_Ac at 0.1 mL/min. LGM fraction was eluted with a propanol-1 linear gradient (5 to 60%) in 100 mM NH_4_Ac for 10 h at the flow rate of 0.15 mL/min. LGM fraction was submitted to a second chloroform/methanol/water (10/10/3) extraction to remove potential presence of lipids and GPI-peptides from GPI-APs. Neutral sugars were detected by the phenolsulfuric method [33]. Molecular size of LGM fraction was analyzed by gel filtration chromatography on a Superdex 75 column (10/300, GE Healthcare, Uppsala, Sweden) after nitrous deamination. The column was eluted with 0.15 M ammonium acetate pH 4 at the flow rate of 0.2 mL/min.

### 2.8. MS Analysis of the GPI Lipid Moiety

The lipid moiety of GPI-anchored molecules was released by nitrous deamination as previously described [13,15]. Briefly, nitrous deamination degraded the glucosamine residue and the released inositolphospholipid (PI) moiety was purified on a small homemade silica gel 60 column (0.063–0.2 mm, 300 µL, Merck, Darmstadt, Germany). Purified PIs were analyzed by mass spectrometry. Samples, dissolved in chloroform/methanol (1/4, *v*/*v*) mixture, were nanoelectrosprayed via a TriVersa NanoMate (Advion, Ithaca, USA) device and analyzed with an Orbitrap Fusion^TM^ Lumos^TM^ mass spectrometer (Thermo Scientific^TM^ Waltham, MA, USA). Mass spectra were recorded in negative mode with a scan range *m*/*z* 300–2000, and 30,000 resolution at *m*/*z* 400. MS/MS spectra were recorded using HCD (High energy Collision Dissociation) with a normalized collision energy set up to 30. GIPCs were analyzed in the same experimental conditions without nitrous deamination degradation.

### 2.9. Statistical Analysis

Results were statistically analyzed by an ANOVA two-way test with a Dunnet’s post-test for conidiation, conidia survival and the composition of alkali-insoluble fraction, using GraphPad Prism 6.0 software with a *p* value below considered as significant (*p* < 0.05 *, *p* < 0.01 **, *p* < 0.001 ***, *p* < 0.0001 ****).

## 3. Results

### 3.1. Construction of the ∆per1 Mutant, Mycelial Growth and Hyphal Morphology

**Figure 2:**
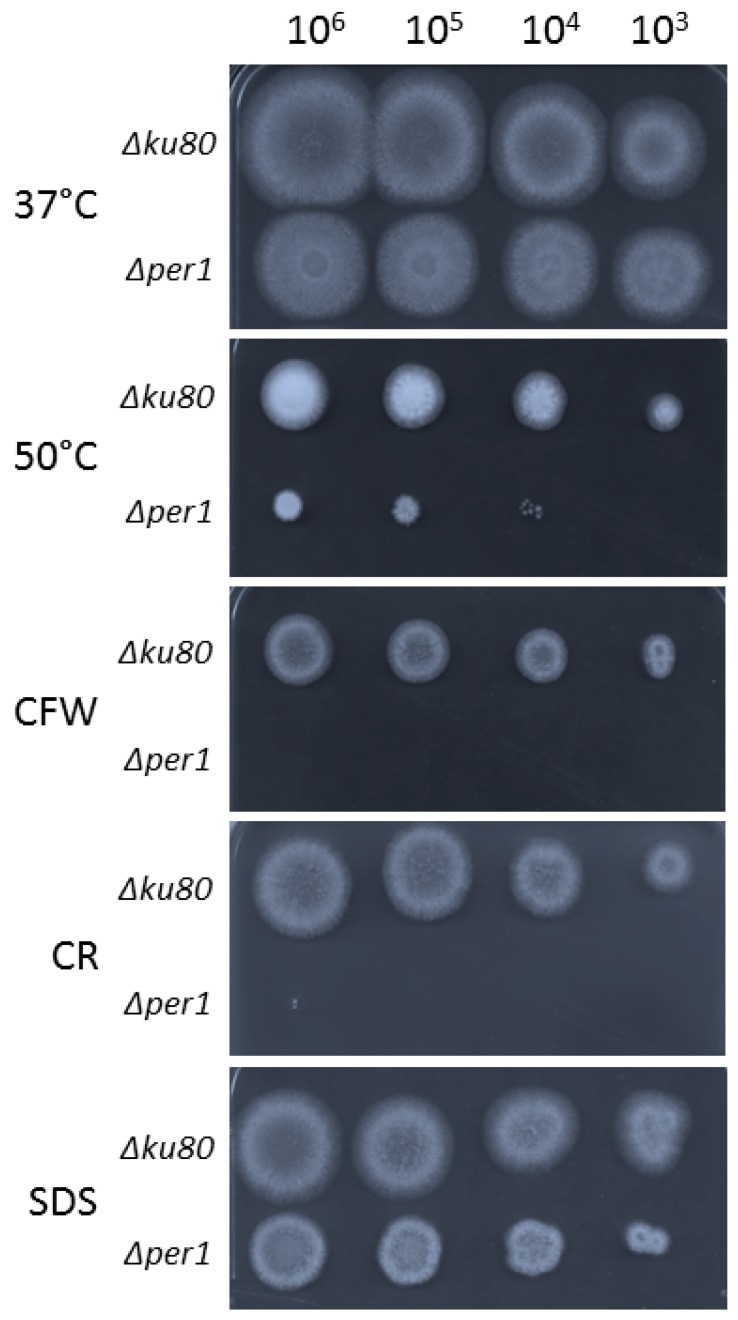
Growth of *PER1* deletion mutant strain on solid medium. Radial growth of the parental strain and *PER1* deletion mutant strain on malt agar medium (48–72 h at 37 °C or 50 °C) with or without calcofluor white (CFW, 40 µg/mL), Congo red (CR, 50 µg/mL), SDS (0.01%).

**Figure 3:**
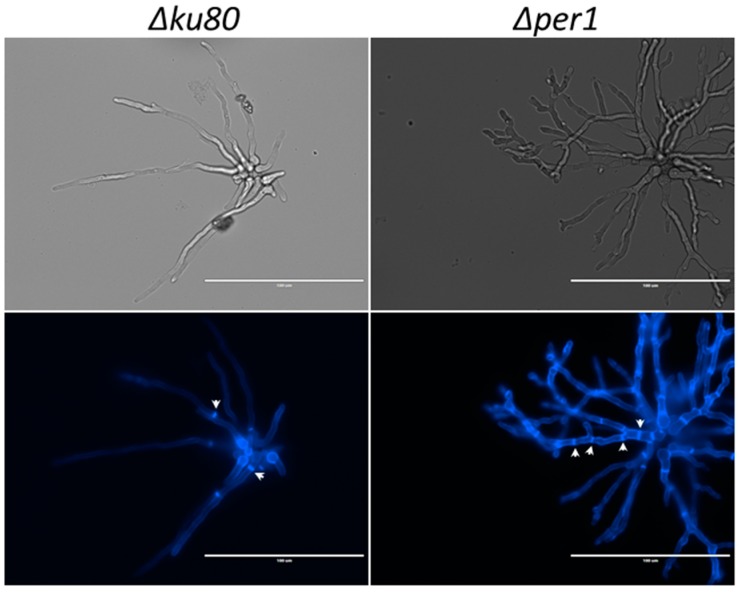
Morphology of the *Δper1* mutant. Microscopic observation of mycelium after 10–13 h at 37 °C in liquid minimum medium. Both bright-field (upper panels) and fluorescence after calcofluor white staining (lower panels) images are shown. Arrows indicate septum location.

The strategy of gene replacement and its validation by Southern blot allowed the isolation of a *∆per1* mutant as shown in Figure S1. On solid malt medium at 37 °C, no difference of radial growth was observed between the *∆per1* mutant and its parental strain (Figure 2). However, the *∆per1* mutant showed a growth defect at 50 °C and in the presence of cell wall interfering dyes such as calcofluor white (CFW) and Congo red (CR). In addition, the sensitivity of the *∆per1* mutant to 0.01% SDS in medium was slightly increased in comparison to the parental strain. Similar growth phenotypes were observed in solid minimum medium (Figure S2), suggesting that the deletion of *PER1* had a stronger impact on the cell wall rather than on membrane organization. In liquid Sabouraud medium at 37 °C, a decrease of 20% of mycelium dried weight was observed for the *∆per1* mutant in comparison with the parental strain (Figure S3). The reduction of mycelial growth was associated with a strong modification of morphology. The *∆per1* mutant produced a hyperbranched hyphae with an increase in the number of septum identified by CFW staining (Figure 3).

### 3.2. Conidiation and Conidial Viability

**Figure 4:**
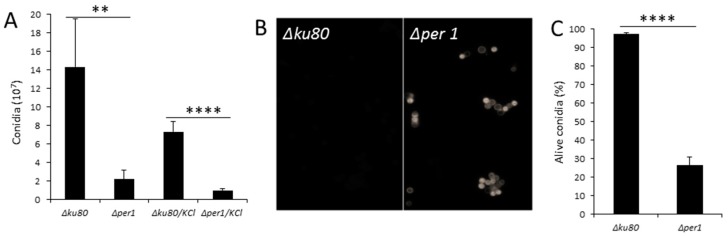
Conidia phenotypes of the *Δper1* strain. (**A**) Conidiation after 1 week on Malt medium; (**B**) FITC staining of 2 weeks-old conidia (10^6^ conidia/mL, acquisition: 1 ms); (**C**) Viability of 40-day-old conidia. (Values are the mean and standard deviation of three different experiments, statistical difference are indicated by ** *p* < 0.01; **** *p* < 0.0001).

The production of conidia by the *∆per1* mutant was reduced 7 times in comparison with the parental strain. The addition of 6% KCl as osmotic stabilizer did not restore a normal conidiation (Figure 4A). The FITC staining showed a strong labelling of *PER1* deficient conidia in comparison with the parental strain, characterizing an increase in permeability of the conidia cell wall. The survival of the *∆per1* mutant conidia was also affected. After 40 days on Malt-agar slant at room temperature, 74% of *∆per1* conidia died whereas less than 5% of the conidia of the parental strain died (Figure 4C).

### 3.3. Localisation of GPI-APs

**Figure 5:**
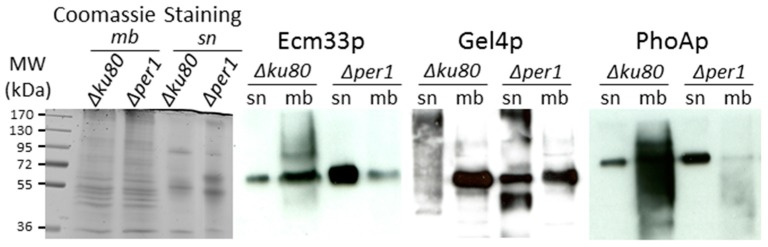
Western blot detection of GPI-APs. Three GPI-APs were analyzed in the total membrane fraction and culture supernatant of parental and and *Δper1* mutants. sn: culture supernatant, mb: membrane extract. (30 µg of protein were loaded by lane).

The localization of GPI-APs in the membrane fraction versus culture medium was analyzed by Western blot using specific antibodies. Three major GPI-APs were analyzed: Ecm33p, Gel4p, and PhoAp [13,38]. In the parental strain, all of these proteins were mainly localized in the membrane fraction (Figure 5). In the *∆per1* mutant, Ecm33p and PhoAp were secreted in the culture supernatant, Gel4p was found in both the culture and membrane fractions. These data showed that the deletion of *PER1* led to a modification of GPI-APs localization with a strong increase in the release of GPI-APs in the culture medium.

### 3.4. Cell Wall Composition

**Figure 6:**
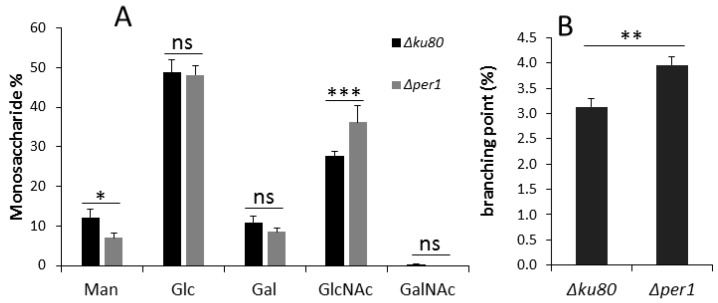
Analysis of the cell wall alkali-insoluble fraction. (**A**) Monosaccharide composition (Man, mannose; Glc, glucose; Gal, galactose; GlcNAc, *N*-acetylglucosamine; GalNAc, *N*-acetylgalactosamine); (**B**) percentage of branching of β-1,3-glucan. (values are the mean and standard deviation of three different experiments, statistical difference are indicated by * *p* < 0.05; ** *p* < 0.01; *** *p* < 0.001; ns, non significative).

Global sugar composition was performed by colorimetric and chromatographic assays. No significant difference was observed in the alkali-soluble fraction between the parental and mutant strains (Table S2). In contrast, the deletion of *PER1* induced an increase of 30% of the GlcNAc amount in the alkali-insoluble fraction (Table S2 and Figure 6), corresponding to an increase of chitin content. The identification and quantification of hexose was performed by GC. The deletion of *PER1* induced a decrease of 34% of mannose amount in the AI fraction (Figure 6), corresponding to a decrease of galactomannan cross-linked to the β-1,3-glucan in the cell wall. Since some β-1,3-glucan remodelase activities have been described as being GPI-anchored, the branching level was investigated using a specific endoglucanase and HPLC analysis [4]. The analysis showed that the deletion of *PER1* induced an increase of 26.6% of branching of β-1,3-glucan (Figure 6).

### 3.5. Analysis of Lipid Moiety of GPI Anchors

**Figure 7:**
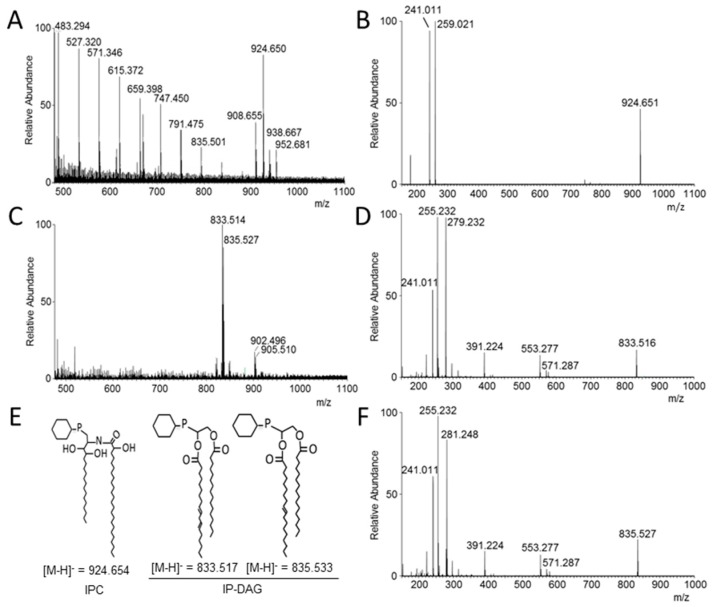
MS data of the lipid moieties of GPI anchors purified from GPI-APs. MS spectra from parental strain (**A**) and the *Δper1* mutant (**C**). MS-MS spectra of the ion *m*/*z* 924.650 from parental strain (**B**) and ions *m*/*z* 833.514 and 835.527 from the *Δper1* mutant (**D**,**F**). Panel (**E**) represents the structure of deduced lipid moieties with their theoretical mass (IPC, inositolphosphoceramide; IP-DAG, Inositolphosphodiacylglycerol). (Major ion fragments in panels B, D and F: *m*/*z* 241.011, Inositol-cyclicphosphate; *m*/*z* 259.021, inositol-phosphate; *m*/*z* 255.232, palmitate (C_16:0_); *m*/*z* 279.232, linoleate (C_18:2_); *m*/*z* 281.248, oleate (C_18:1_)).

In order to analyze the lipid anchor in the *∆per1* mutant, GPI-APs and LGM were purified by chromatography. Two anion exchange chromatographic steps allowed the isolation of a GPI-AP fraction. Western blot detection of protein bands using an anti-CRD antibody after PI-PLC digestion showed that the GPI-AP fraction contained GPI-APs with their whole GPI-anchor (Figure S4), which was essential to the investigation of the structure of the lipid moiety of GPI anchors. Lipid moiety was released by nitrous deamination and characterized by mass spectrometry. MS data from *∆ku80* strain showed the presence of two types of ions (Figure 7A). The first group of ions ranging from *m*/*z* 483.294 to 835.501 corresponds to a contamination with Triton X100 as indicated by the repetitive units of C_2_OH_4_ (44.026 Da). The second group of ions from *m*/*z* 908.655 to 952.681 corresponds to the inositol-phosphate-ceramide moieties already characterized in *A. fumigatus* [13]. The MS/MS analysis of the most abundant major ion at *m*/*z* 924.650 led to two major fragments at *m*/*z* 241.011 [inositol-1,2cyclicphosphate]^−^ and *m*/*z* 259.021 [inositol-phosphate]^−^. These two diagnostic ions and the absence of a fragment ion arising from an acyl chain characterized the presence of a ceramide group [39]. This confirms that the ion at *m*/*z* 924.650 corresponds to an inositolphosphoceramide (IPC) composed of the C_18_-phytosphingosine and 2-monohydroxylated-C_24:0_ fatty acid as previously described [13]. The ions [M-H]^−^ at *m*/*z* 908.655, 938.667 and 952.681 correspond respectively to different forms of the ceramide: ceramide where an hydroxyl function of the fatty acid has been replaced by an hydrogen, and the addition of one or two methylene groups in the aliphatic chain. MS data from the lipid moiety isolated from the *∆per1* mutant were totally different with the absence of the ion [M-H]^−^ *m*/*z* 924.650 and the presence of the main ions at *m*/*z* 833.514 and 835.527 (Figure 7C). The fragmentation of the ion at *m*/*z* 833.514 gave three main ions at *m*/*z* 241.011 [inositol-1,2cyclicphosphate]^−^, 255.232 [palmitate]^−^ and 279.232 [linoleate]^−^. Based on the fragmentation of phosphoinositides, these MS-MS data showed the presence of a dicacylglycerol linked to a inositolphosphate group [39,40]. The fragmentation of the ion at *m*/*z* 835.527 showed that it has the same structure where the linoleate is substituted by an oleate (formation of a fragment ion at 281.248 instead of 279.232) (Figure 7E,F). These MS data showed that the deletion of *PER1* has blocked the lipid remodeling of GPI in *A. fumigatus* at the first step leading to the sole presence of a diacylglycerol as the lipid moiety of the GPI anchor.

**Figure 8:**
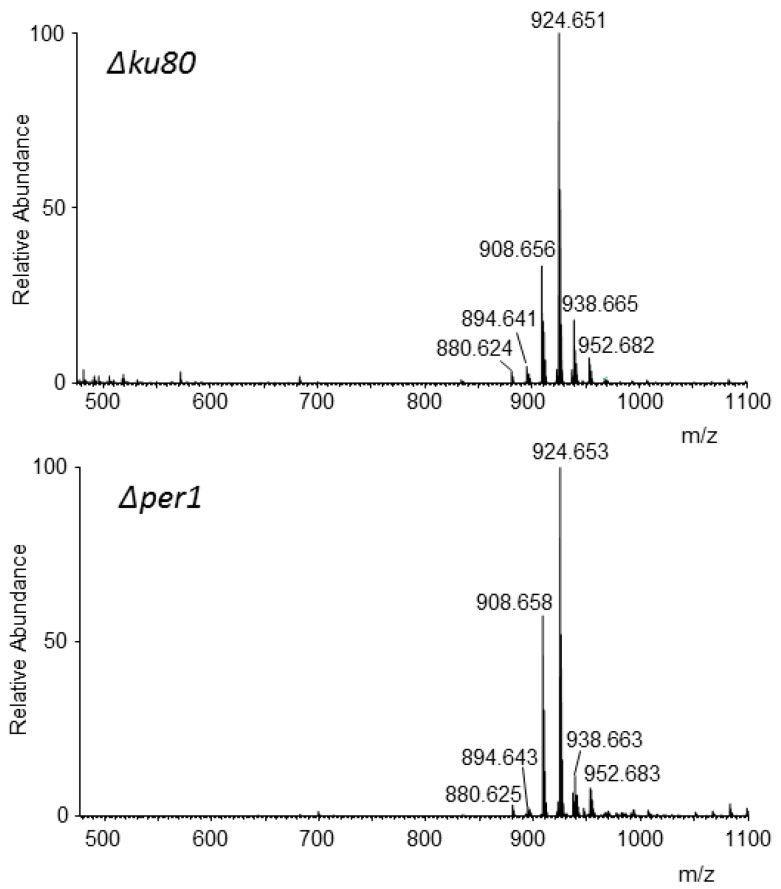
MS data of lipid moieties of the purified lipogalactomannan LGM from parental (*Δku80*) and *Δper1* mutant strains. (Major ions: *m*/*z* 924.651, inositolphosphoceramide (IPC) composed of a C_18_-phytosphingosine and 2-OH-C_24:0_; *m*/*z* 908.656, IPC composed of C_18_-phytosphingosine and C_24:0_).

Purified LGM fractions from parental and *Δper1* mutant strains contained only mannose and galactose as hexose and were eluted as a 30 kDa polymer by gel filtration chromatography (Figure S5). The lipid moiety of the LGM was analyzed by the same methodology. MS data of LGM from *∆ku80* and *∆per1* mutants were strictly similar with the presence of ions [M-H]^−^ at *m*/*z* 908.656, 924.651, 938.655, and 952.682 (Figure 8). Fragmentation of all of these ions induced the formation of two major fragment ions at *m*/*z* 241.011 and 259.021 characterizing IPC structures as previously described for GPI from GPI-APs and LGM from the *∆ku80* parental strain [13,15] (Figure S6).

## 4. Discussion

Lipid remodeling of GPI in fungi has been well studied in *S. cerevisiae* where its sequential steps occur after the transfer of the target proteins on to the GPI-anchor in the ER. In this study, we have investigated the function of Per1p in the filamentous fungus *A. fumigatus*. MS data of the lipid moiety of GPI-APs in the *Δper1* mutant showed the absence of ceramide but the presence of a diacylglycerol mainly composed of palmitate and oleate or palmitate and linoleate fatty acids, showing that Per1p plays the same function in yeast and in *A. fumigatus*. In yeast, two models have been proposed to describe the lipid remodeling pathway: the main sequential pathway and a minor alternative pathway in which Cwh43p may use the PI containing a diacylglycerol (pG2) or a monoacylglycerol lysopG2 (lysoPI) as substrate (Figure 1B). Indeed, in *Δper1* and *Δgup1* mutants, a small fraction of GPI contained an IPC structure [24,26]. In *A. fumigatus*, MS data did not allow detection of ion mass at *m*/*z* 924.654 from GPI-APs of the *Δper1* mutant, suggesting the absence of an alternative pathway in *A. fumigatus*. 

This study has shown that *PER1* is required for the normal growth, conidiation, viability, and permeability of resting conidia and cell wall assembly in *A. fumigatus*, showing that GPI lipid remodeling in filamentous fungi is essential for cellular trafficking of GPI-APs toward the plasma membrane and for their biological function as described in yeast. In addition, it has been shown that the *∆per1* mutant was avirulent in a mouse model of invasive aspergillosis and that the deletion of *PER1* altered the exposure of PAMPS in *A. fumigatus* by increasing the β-1,3-glucan detection at the fungal cell surface [28]. Accordingly, our biochemical data have shown specific modifications in the cell wall organization of the *Δper1* mutant. Despite having the same global amount of β-1,3-glucan, the deletion of *PER1* induced an increase in chitin amount and a decrease in cell wall cross-linked GM. A higher amount of chitin is usually associated with a compensatory mechanism in fungi that was observed in many cell wall mutants. In contrast, the alteration of β-1,3-glucan branching and of the content of alkali-insoluble galactomannan should be a direct effect of the absence of Per1p. Elongation and branching of cell wall β-1,3-glucans are catalyzed by specific GPI-anchored β-1,3-glucanosyltransferase activities [4,6,7,8]. GPI-anchored Dfgp members are required for the cross-linking of the galactomannan onto β-1,3-glucan [11]. The *PER1* deletion led to a partial misslocation of GPI-APs (Figure 5). In particular, Gel4p—which is an essential β-1,3-glucanosyltransferase in *A. fumigatus*—became released in the culture medium. In yeast, the interruption of the GPI lipid remodeling leads to the alteration of GPI-APs integration in detergent-resistant microdomains (DRM) and their cellular trafficking [14,15]. The GPI-anchor was not required in vitro for enzyme activity [4,6], the structure of the lipid of GPI-APs was essential for the correct localization at the outlet layer of the plasma membrane, where the β-1,3-glucans are synthesized and may be required for the cooperation in a close environment between enzymes involved in cell wall β-1,3-glucan remodeling.

Among membrane-bound molecules produced by *A. fumigatus*, the LGM also contains a GPI-anchor similar to those of GPI-APs [15]. The *∆per1* mutant was still producing this LGM and its lipid anchor contains an IPC identical to the one of the parental strain, showing that the deletion of *PER1* has no effect on the lipid anchoring of GM and the biosynthesis of the LGM is independent of the GPI biosynthetic pathway of the GPI-anchor. IPC structures are described in fungal specific glycosphingolipids also called GIPC. Two types of GIPC are produced in *A. fumigatus*, acidic and zwitterionic GIPC [37,41]. GIPCs from the *∆per1* mutant were analyzed by MS (Figure S7). The MS data of the GIPC fractions from *∆ku80* and *∆per1* were similar and corresponded to the presence of 3 or 4 hexose residues with or without a choline-phosphate group linked to an IPC (Figure S7) [37]. The biosynthesis of these GIPCs starts with the addition of glucosamine or mannose residue onto the inositol ring of the IPC [42,43]. These data suggest that LGM may be also synthetized onto an IPC anchor. The inactivation of glycosyltransferases (mannosyltransferase and *N*-acetylglucosaminyltransferase), required for the GIPC synthesis has no effect on the GM synthesis [42,43] showing that GM synthesis is also independent of GIPC synthesis. The LGM is composed of a main chain of α-mannoside residues linked to a glucosamine-IPC anchor with a small side chain of galactofuranose residues. The addition of galactofuranose in *A. fumigatus* has already been described [44,45,46,47]. The polymerization of the mannan chain and its anchoring to IPC remain totally unknown. In yeast, elongation of N-mannan chains (up to 150 residues of mannose) occurs in Golgi by the sequential activities of mannosyltransferase [48]. Recently, in *A. fumigatus*, a multiple mutant deficient in 11 mannosyltransferase activities, orthologous to those involved in N-mannan in yeast has been described [49]. Despite the large number of deletions, the vegetative growth of this multiple mutant was similar to the parental strain and no modification of GM structure was observed suggesting that the GM follows an independent and specific biosynthetic pathway. The identification of glycosyltransferases in this GM pathway is essential to the understanding of the biological functions of the GM in filamentous fungi. The genome of *A. fumigatus* contains at least 35 genes coding for putative α-mannosyltransferases. A number of them are involved in GPI, *O*- and *N*-glycosylation, and mannan elongation. Those required for the GM polymerization remain unknown. A mannosyltransferase activity—able to transfer a mannose residue onto a GlcN-PI—independent of the GPI pathway has been detected in vitro in *A. fumigatus* [18], but its involvement in GM synthesis still remains to be elucidated. Biochemical and molecular approaches to understanding the function of all expressed mannosyltransferases in *A. fumigatus* are currently in progress.

## Supplementary Materials

The following are available online at http://www.mdpi.com/2309-608X/4/1/19/s1.
