# Supplementary material for: Glycosylphosphatidylinositol Anchors from Galactomannan and GPI-Anchored Protein Are Synthesized by Distinct Pathways in Aspergillus fumigatus
Source: J Fungi (Basel). 2018 Jan 23;4(1):19. doi: 10.3390/jof4010019 (PMC5872322; doi:10.3390/jof4010019)
Supplement: Supplementary file 1 [file jof-04-00019-s001.pdf]

Glycosylphosphatidylinositol Anchors from Galactomannan and GPI-Anchored Protein Are Synthesized by Distinct Pathways in *Aspergillus fumigatus*

Jizhou Li [1], Isabelle Mouyna [1], Christine Henry [1], Frédérique Moyrand [2], Christian Malosse [3], Julia Chamot-Rooke [3], Guilhem Janbon [2], Jean-Paul Latgé [1] and Thierry Fontaine [1,*]

[1] Unité des Aspergillus, 25 rue du Docteur Roux, Institut Pasteur, 25 rue du Docteur Roux, 75015 Paris, France; lee19910503@gmail.com (J.L.); imouyna@pasteur.fr (I.M.); chenry@pasteur.fr (C.H.); jplatge@pasteur.fr (J.-P.L.); thierry.fontaine@pasteur.fr (T.F.)

[2] Unité de Biologie des ARN des Pathogènes Fongiques, Institut Pasteur, 25 rue du Docteur Roux, 75015 Paris, France; frederique.moyrand@pasteur.fr (F.M.); guilhem.janbon@pasteur.fr (G.J.)

[3] Unité de Spectrométrie de Masse pour la Biologie, Institut Pasteur, CNRS USR 2000, 28 rue du Docteur Roux, 75015 Paris, France; cmalosse@pasteur.fr (C.M.); julia.chamot-rooke@pasteur.fr (J.C.-R.)

* Correspondence: thierry.fontaine@pasteur.fr; Tel.: +33-145-688-358

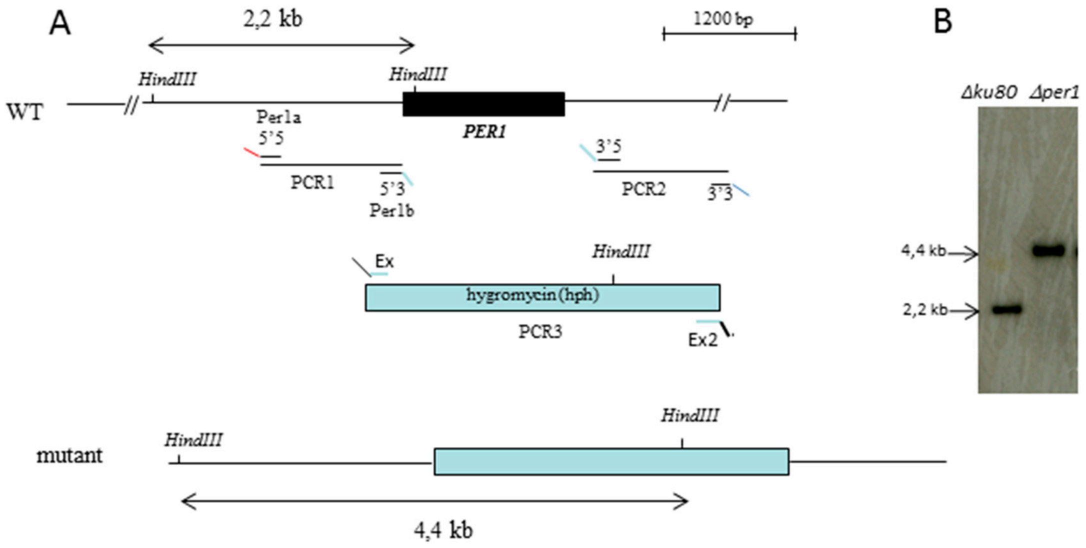

Figure S1. (**A**) Construction of Δ*per1* mutant by PCR fusion. (**B**) Southern blot analysis: Genomic DNA has been digested by *Hind*III and hybridized with PCR1 probe amplified with primers Per1a-Per1b.

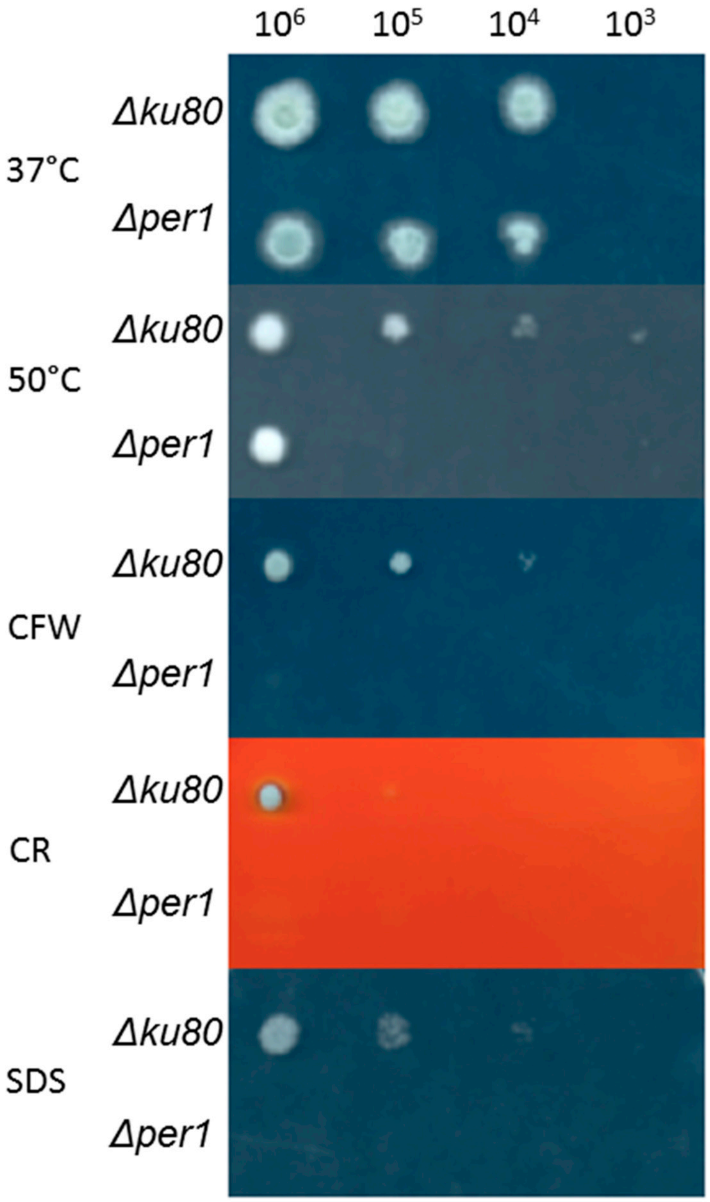

Figure S2. Growth of PER1 deletion mutant strain on solid minimum medium. Radial growth of the parental strain and PER1 deletion mutant strain on malt agar medium (48–72 h at 37 °C or 50 °C) with or without calcofluor white (CFW, 40 µg/mL), Congo red (CR, 50 µg/mL), SDS (0.01%).

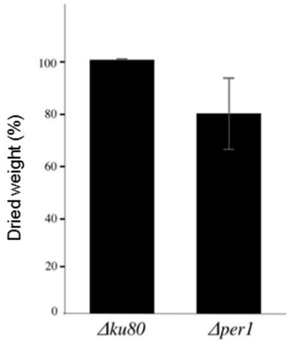

Figure S3. Growth of PER1 deletion mutant strain on Liquid Sabouraud medium. Growth was estimated as the measure of the dried weight of biomass.

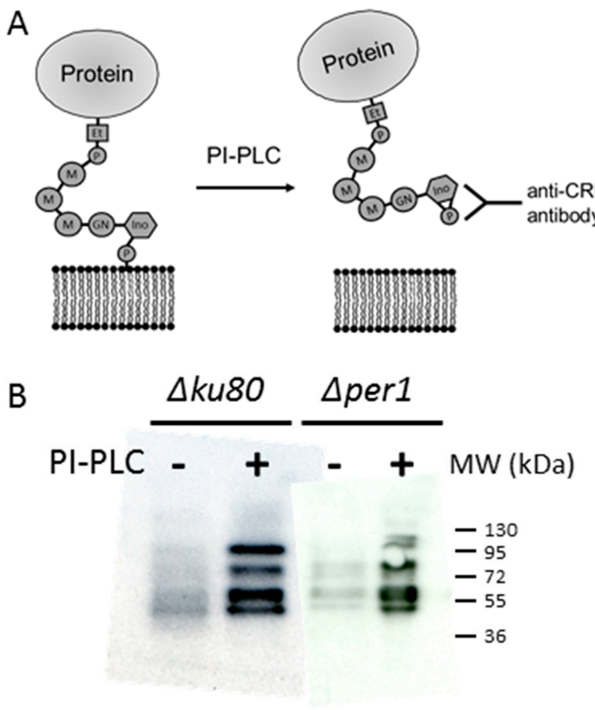

Figure S4. Detection of purified GPI-Aps. (**A**) Scheme of detection using a PI-phospholipase C and an anti-CRD antiboby. (**B**) Western blot of GPI-APs fraction purified from parental (*Δku80*) and *Δper1* mutant strains.

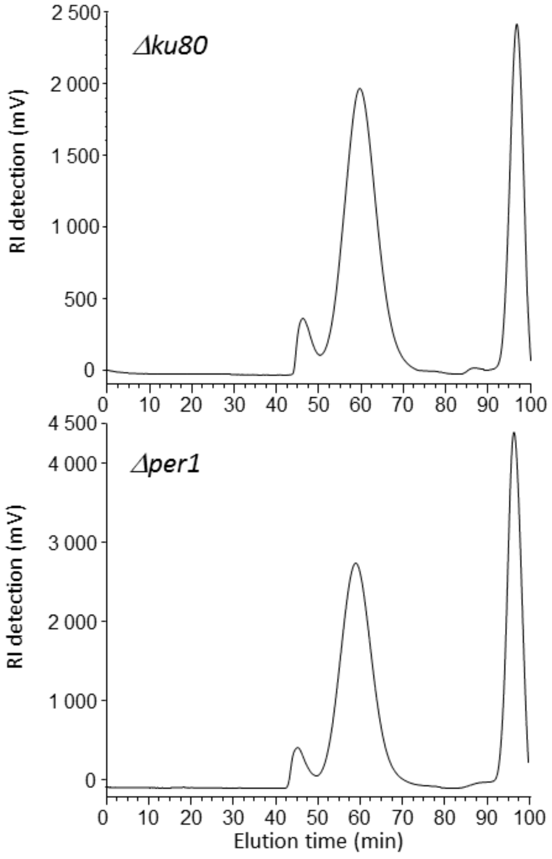

Figure S5. Gel filtration chromatography on Superdex 75 of purified LGM fraction from *Δku80* and *Δper1* strains. Prior to the analysis, LGM was submitted to a nitrous deamination. Products were detected by a RI detector.

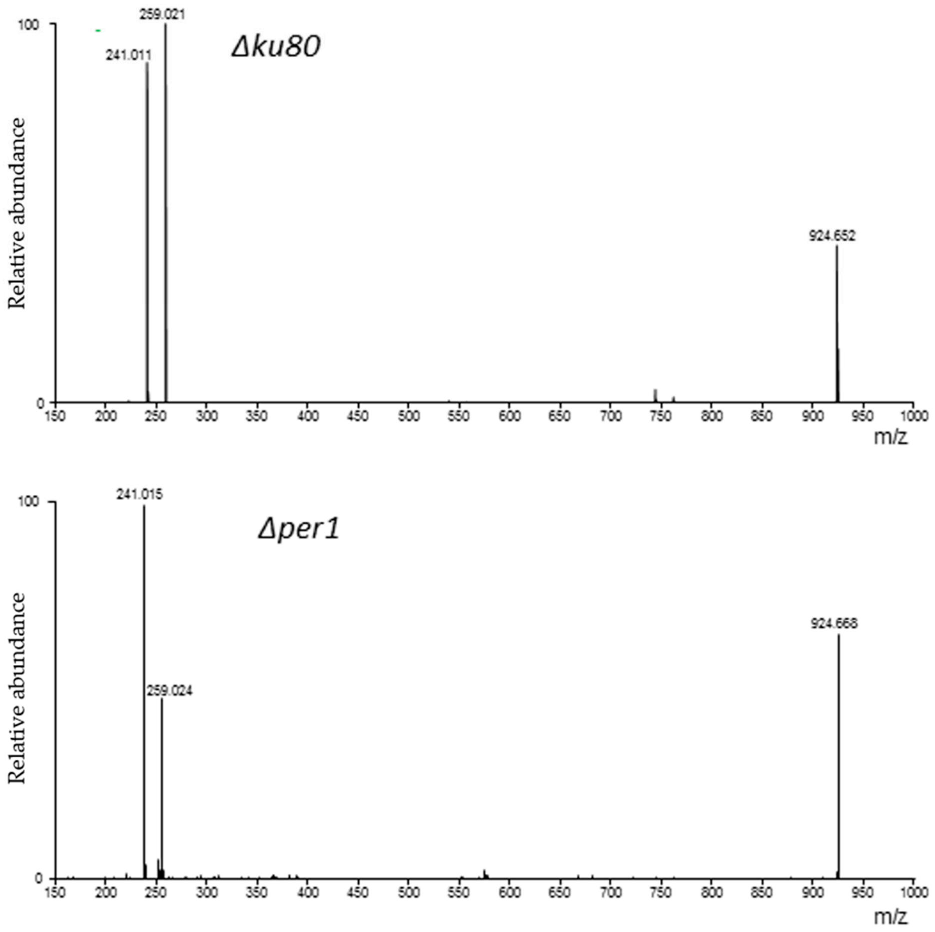

Figure S6. MS-MS spectra of the ion *m/z* 924.650 of the lipid anchor of LGM isolated from the parental strain (*Δku80*) and from the mutant strain (*Δper1*).

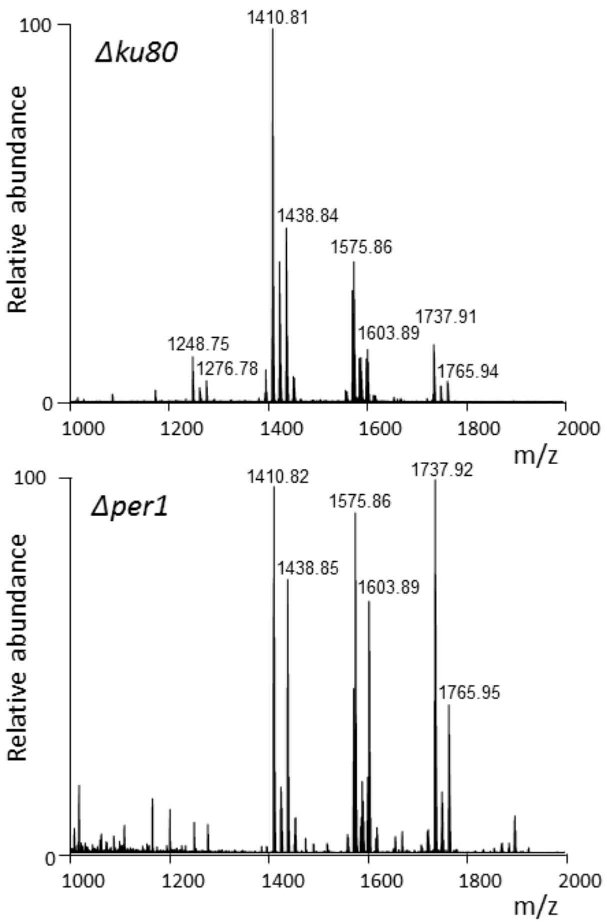

Figure S7. MS spectra of GIPC fraction isolated from parental (*Δku80*) and *Δper1* mutant strains. The MS data of GIPC fractions from *Δku80* and *Δper1* were similar with the presence of characteristic ions [M-H]$^-$ at m/z 1410.8, 1572.8, 1575.9 and 1737.9 corresponding to the presence of 3 or 4 hexose residues with or without a choline-phosphate group linked to a IPC [38].

Table S1. Primers used in the paper.

Name	Sequence
Primers used to construct the *per1Δ::HYG* deletion cassette	
AFUB_006580-3′3	GCGGATAACAATTTCACACAGGAAACAGCGATTATGGCTCGCAGTGACC
AFUB_006580-3′5	CTCCTTCAATATCATCTTCTGTCTCCAACACGCGTTCCCAGAATGATGTCTCAAGCCGC
AFUB_006580-5′5	GTAACGCCAGGGTTTTCCCAGTCACGACGCTCATGAATGCCTTAGCACGG
AFUB_006580-5′3	ATCCACTTAACGTTACTGAAATCTCCTTCACCATGGTGCTAATGGAGTGG
AFUB_006580-Ex	ATCCACTTAACGTTACTGAAATCTCCTTCACCATGGTGCTAATGGAGTGG
AFUB_006580-Ex2	AACGAAGTGTCAGCATCGAGAG
AFUB_006580-MKRr	GGGAACGCGTGTTGGAGACAGAAGATGATATTGAAGGAG
AFUB_006580-MKRf	GAAGGAGATTTCAGTAACGTTAAGTGGAT
Primers used to construct the pNE478 plasmid	
GFPf	GAAGGAGATTTCAGTAACGTTAAGTGGATATGGTGAGCAAGGGCGAGGA
GFPr	AGATCTGGATCCTTTACTTGTACAGCTCGTCC
HygF	AGATCTGTCCAATTGCTTCCGATCTGG
HygR	GTTGGAGACAGAAGATGATATTGAAGGAGCGCGGCCGCGATGAATGTGTGTCCTGTAGGC
Primers used to do the DIG probe	
Per1a	CATGAATGCCTTAGCACGG
Per1b	CACTCCATTAGCACCATG

Table S2. Global sugar composition of cell wall AI and AS fractions (%).

	%	%
Alkali-insoluble fraction	*Δku80*	*Δper1*
Mannose	12.16 +/− 2.19	7.1 +/− 1.01
Glucose	48.81 +/− 3.25	48.17 +/− 2.26
Galactose	10.96 +/− 1.6	8.52 +/− 0.92
GlcNac	27.71 +/− 1.2	36.2 +/− 4.17
GalNAc	0.35 +/− 0.07	0
Alkali-soluble fraction		
Mannose	3.41 +/− 1.21	3.44 +/− 1.59
Glucose	81.5 +/− 3.48	82.6 +/− 4.74
Galactose	9.3 +/− 1.93	7.91 +/− 3.77
GlcNAc	0	0
GalNAc	5.8 +/− 1.14	6.05 +/− 0.64
